# A phenylthiazole derivative demonstrates efficacy on treatment of the cryptococcosis & candidiasis in animal models

**DOI:** 10.4155/fsoa-2018-0001

**Published:** 2018-04-25

**Authors:** Nívea P Sá, Caroline M Lima, Julliana R A dos Santos, Marliete C Costa, Patrícia P de Barros, Juliana C Junqueira, Jéssica A Vaz, Renata B Oliveira, Beth B Fuchs, Eleftherios Mylonakis, Carlos A Rosa, Daniel A Santos, Susana Johann

**Affiliations:** Department of Microbiology, Institute of Biological Sciences, Universidade Federal De Minas Gerais, Belo Horizonte, MG, Brazil; 2 Postgraduate Program, Universidade CEUMA (UNICEUMA), São Luís, MA, Brazil; 3 Department of Biosciences & Oral Diagnosis, Institute of Science & Technology, Univ Estadual de São Paulo, São José dos Campos, São Paulo, Brazil; 4 Department of Pharmaceutical Products, Faculdade de Farmácia, Universidade Federal de Minas Gerais, Belo Horizonte, MG, Brazil; 5 Division of Infectious Diseases, Rhode Island Hospital, Alpert Medical School & Brown University, Providence, RI, 02903, USA

**Keywords:** antifungal, *Candida albicans*, *Cryptococcus* spp., *Galleria mellonella*, mice, thiazole, virulence

## Abstract

**Aim:**

In this work we test 2-(2-(cyclohexylmethylene)hydrazinyl)-4-phenylthiazole (CHT) against *Cryptococcus* spp. and *Candida albicans*.

**Methods:**

The ability of CHT to act in biofilm and also to interfere with *C. albicans* adhesion was evaluated, as well as the efficiency of the CHT in cryptococcosis and candidiasis invertebrate and murine models.

**Results & conclusion:**

In the present work we verified that CHT is found to inhibit *Cryptococcus* and *C. albicans* affecting biofilm in both and inhibited adhesion of *Candida* to human buccal cells. When we evaluated *in vivo*, CHT prolonged survival of *Galleria mellonella* after infections with *Cryptococcusgattii*, *Cryptococcusneoformans* or *C. albicans* and promoted a reduction in the fungal burden to the organs in the murine models. These results demonstrate CHT therapeutic potential.

## Lay abstract

*Candida* spp. and *Cryptococcus* spp. cause thousands of deaths each year. In general, antifungal drugs have several limitations to their use, and there are a limited number of these drugs available to be used in the treatments of fungal diseases. This work contributes to the search for new antifungal drugs for the treatment of candidiasis and cryptococcosis, aiming in the future, after all necessary tests, to serve as a basis for the production of drugs that could be used in the treatment of patients with these fungal diseases.

## Graphical abstract

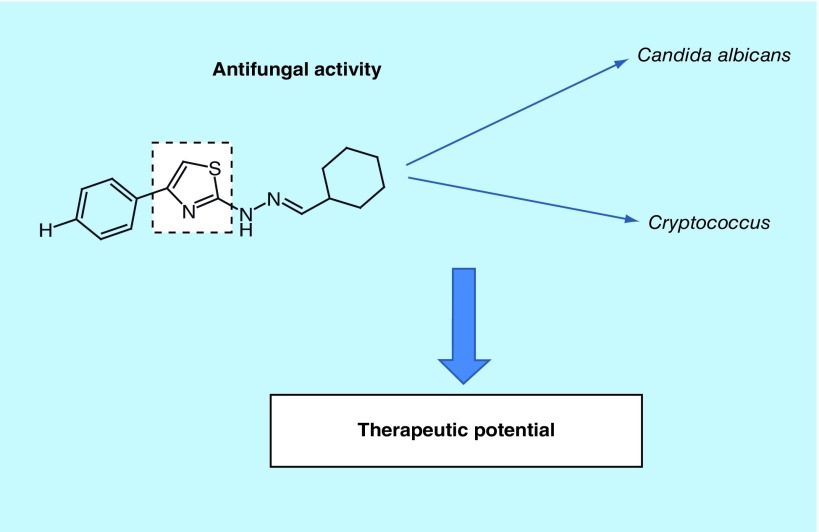

Human body temperature provides a protective thermal barrier against the majority of environmental fungal species; however, about 300 species may cause disease in humans [1]. The most common pathogens that cause almost 90% of deaths due to fungal disease are *Candida* spp., *Aspergillus* spp., and *Cryptococcus* spp. [2,3]. We will focus our discussion to the two pathogens: *Candida albicans* and *Cryptococcus*.

Cryptococcosis is responsible for more than one million cases and about 650,000 deaths per year worldwide, as an infectious disease with a universal geographical distribution, but clinical cases are more frequent in the Americas and Africa [3,4]. Cryptococcal infections occur by inhalation of the infectious propagules [5]. The opportunistic pathogen *Cryptococcus neoformans* is associated with immunodepression, while infection of immunocompetent hosts occurs primarily with *Cryptococcus gattii* [5,6]. Both forms of cryptococcosis, opportunistic and primary, are capable of causing severe and fatal meningoencephalitis, while the infection may involve organs such as the lung, skin, bones or kidneys [7].

*C. albicans*, the primary agent of candidiasis, is a commensal in healthy humans and can cause infections that are associated with staggeringly high mortality rates [8]. An estimated 24–70% of healthy people above 1 year of age are colonized by *Candida* and everyone is temporarily colonized at least once during their lifetime [9].

Amphotericin B, fluconazole and 5-fluorocytosine are antifungal agents most often used to treat patients with disseminated candidiasis and cryptococcosis [10]. Amphotericin B plus flucytosine combination is the treatment of choice during the induction phase in the treatment of cryptococcosis [11]. Unfortunately, amphotericin B is nephrotoxic, while flucytosine is not available in many countries and it is associated with bone marrow suppression and liver toxicity [11,12]. Maintenance therapy is supported by fluconazole, one of the major antifungal agents used in the treatment of cryptococcosis and disseminated candidiasis, as it has high efficacy and low toxicity [13]. However, one of the problems with fluconazole is the increasing incidence of fungal resistance [14,15].

Thus, a need remains to increase the drug arsenal of antifungal compounds. Our group has created some thiazoles derivatives with antifungal efficacy [16]. In the present work we evaluated the therapeutic potential of 2-(2-(cyclohexylmethylene)hydrazinyl)-4-phenylthiazole (CHT) against some of the prominent agents that lead to cryptococcosis and candidiasis.

## Materials & methods

### Compound

**Figure 1.**
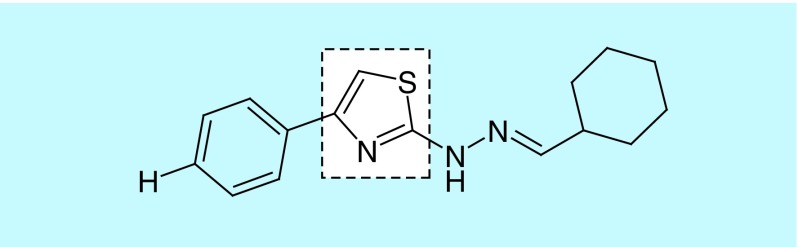
**Chemical structure of 2-(2-(cyclohexylmethylene)hydrazinyl)-4-phenylthiazole, highlighting the thiazole portion.**

Thiazole compound CHT was synthesized according to methodology previously described by Sá *et al*. [16], previously designated as 1d by these authors (Figure 1).

### Fungal strains

*C. neoformans* ATCC24067 (AFLP2/VNIV) (= *Cryptococcus deneoformans*, according name proposed by Hagen *et al*. [17]) was obtained from the Culture Collection of the University of Georgia (GA, USA) and *C. gattii* L27/01 (VNII by PCR-fingerprinting using [GTG]5 primer, Unpublished Data; = *Cryptococcus deuterogattii*, according name proposed by Hagen *et al*. [17]) from the Culture Collection of Microorganisms and Cells of the Universidade Federal of Minas Gerais under code UFMG-CM-Y6141 (Belo Horizonte, MG, Brazil). *C. albicans* SC5314, ATCC18804 and 10 *C. albicans* clinical isolates (UFMG-CM-Y6071, UFMG-CM-Y6072, UFMG-CM-Y6073, UFMG-CM-Y6074, UFMG-CM-Y6075, UFMG-CM-Y6076, UFMG-CM-Y6077, UFMG-CM-Y6080, UFMG-CM-Y6081 and UFMG-CM-Y6082), also deposited in the Culture Collection of Microorganisms and Cells of the Universidade Federal of Minas Gerais, were used in this study. Yeasts were grown on Sabouraud dextrose medium (Himedia, Mumbai, India) for 24 and 48 h for *C. albicans* and *Cryptococcus* spp., respectively, at 37°C prior to the experiments. All yeast strains were stored in glycerol at -80 °C.

### *In vitro* susceptibility of *C. albicans*

Broth microdilution testing was performed in accordance with the guidelines in the Clinical & Laboratory Standards Institute (CLSI) document M27-A3 [18]. Susceptibility was determined by the microbroth dilution method. Briefly, *C. albicans* strains were suspended in phosphate-buffered saline (PBS). CHT was dissolved in DMSO (Sigma, MO, USA) and diluted in RPMI 1640 medium supplemented with L-glutamine and buffered to pH 7.0 with 0.165 M morpholine propanesulfonic acid (Sigma). The compounds were tested at concentrations of 0.125–64 mg/l. As growth and sterility controls, RPMI alone was used, without the addition of the compound and solvent. In addition, a control for the toxicity of the solvent, a culture was inoculated with DMSO. Fluconazole (Sigma) and amphotericin B (Sigma) were included as positive antifungal controls and were tested at concentrations of 0.125–64 mg/l. The plates were incubated at 35 °C for 48 h. Three independent experiments were performed in duplicate.

### Antibiofilm activity

#### C. *albicans* biofilm

An inoculum of *C. albicans* SC5314 was adjusted to 10^6^ cells/ml in RPMI medium and 2 ml of the inoculum was distributed in 24-well microplates containing sterile silicone pads. The microplates were incubated at 37°C under agitation of 80× g. At 24 and 48 h, the medium was removed and the wells were washed two-times with PBS, then 2 ml of RPMI 1640 medium was added to the wells. At 72 h the plates were washed again and 2 ml of RPMI containing CHT in concentrations equal to the MIC were added to the biofilms. Plates were incubated for 24 h then washed with PBS and the silicone pads were placed at room temperature to dry before measuring the biofilm dry weight [19]. Six replicates were made in two independent experiments.

### Reduction assay with XTT in biofilm of *Cryptococcus*

Evaluation of *Cryptococcus* biofilm was performed according to Martinez & Casadevall [20] with modifications. *C. gattii* L27/01 and *C. neoformans* ATCC24067 were grown in Sabouraud dextrose broth for 24 h at 37°C with agitation at 120× *g*. Cells were then collected by centrifugation, washed twice with PBS, and suspended at 10^7^ cells/ml in minimal medium. One hundred microliters of the suspension were added into wells of 96-well microplates and incubated at 37°C. After 24 h, the wells containing biofilms were washed three-times with 0.05% Tween 20 (Sigma-Aldrich, MO, USA) in Tris-buffered saline to remove nonadhered cryptococcal cells. Then, we added 100 μl of minimal medium and incubated the plates for another 24 h. Subsequently, the supernatant was removed and the plates were washed, and after that plates were replaced with medium containing CHT compound according to MIC values (1 mg/l) to both *Cryptococcus* strains previously determined by Sá *et al*. [16] for 24 h.

After this period the plates were again washed in order to remove cells not adhered to the biofilm, and metabolic activity of the attached cells was measured with 2,3-bis(2-methoxy-4-nitro-5-sulfophenyl)-5-((phenylamino)carbonyl)-2H-tetrazolium hydroxide (XTT, Sigma) according with Meshulam *et al*. [21]. All assays were carried out in ten replicates.

#### Inhibition of C. albicans adhesion in human buccal epithelial cell

A 1 ml suspension of 10^6^ cells/ml of the 12 *C. albicans* isolates was added to 4 ml of  Saboraud Dextrose Broth(SDB) containing CHT or fluconazole at concentrations equal to the MIC, and a positive control was prepared containing medium without drug. The samples were incubated at 37°C for 1 h with agitation at 120× *g*. After the treatment period, cells were washed twice with PBS before suspension in 3 ml of PBS.

The method described by Kimura and Pearsall [22] with modifications was used to test cell adhesion. Human buccal epithelial cells (HBEC) from healthy adult volunteers were collected in the morning. The HBEC suspension was washed four-times in PBS with centrifugation at 3000 × *g* for 10 min and resuspended in PBS, at a final concentration of 10^5^ cells/ml. Then, 0.5 ml of HBEC suspension and 0.5 ml of yeast suspension were mixed gently and incubated on a rotary shaker at 37°C for 1 h. At the end of this period, cells were filtered on polycarbonate filter with pore size 12 μm and washed with 100 ml of PBS to remove non-adherent yeast cells. After, the filter was removed and put on a glass slide. Preparations were air dried, fixed with heat, and stained with crystal violet. Yeasts adherent to the buccal cells were counted under a light microscope (40×). In each slide, we examined 50 HBEC and counted the number of associated fungal cells.

### Survival curve of *Galleria mellonella*

For the model of *C. albicans* infection in *G. mellonella* studies were performed as previously described by Fuchs *et al*. [23]. *G. mellonella* (Vanderhorst Wholesale, OH, USA) in the final larval stage was stored in the dark and used within 7 days from shipment. In addition, tests with *Cryptococcus* spp. had been performed using *G. mellonella* in the final larval stage and studies were performed as previously described by Mylonakis *et al*. [24]. *G. mellonella* were obtained from Laboratory of Microbiology and Immunology of Univ Estadual Paulista (Unesp, São José dos Campos, São Paulo, Brazil).

*C. albicans* SC5314 was cultured in YPD broth (1% yeast extract, 2% bacto-peptone, 2% dextrose) at 30°C overnight and *C. gattii* L27/01 and *C. neoformans* ATCC24067 were prepared by culturing the cells in YPD broth at 37°C for 48 h and then all yeasts were washed three-times with PBS. Yeast cells were counted using a hemocytometer. A Hamilton syringe was used to inject yeasts in a volume of 10 μl into the hemocele of each larva via the last left proleg. Each larva received 10 μl containing 10^6^ cells/larva of *C. albicans* and *C. neoformans* and 10^7^ cells/larvae of *C. gattii* into the hemocele.

Experimental groups received 10 μl of CHT solubilized in a vehicle (10 mg/kg) and injected at the last right proleg. Two control groups were included in all experiments: one group was inoculated with PBS to account for any killing due to physical trauma, and the other received no injection as a control for general viability. After injections, larvae were incubated in Petri plates at 37°C and monitored for survival daily.

### Murine models

#### Animals

C57BL/6 mice, approximately 6–8 weeks in age and 20–25 g were supplied by the Biological Center of the Federal University of Minas Gerais (Cebio, UFMG, Belo Horizonte, Minas Gerais, Brazil). Male mice were used in the cryptococcosis model and female in the systemic candidiasis model. Tests in the animal models were in accordance with the Ethics Committee for Animal Experimentation (CEUA/UFMG), protocol n° 221/2013.

#### Cryptococcosis

The animals were infected intratracheally with 10^5^ yeasts of *C. gattii* L27/01 in sterile saline (NaCl 0.85%). After 24 h of infection, the mice were divided into five experimental groups (n = 6 animals/group) as following: control not infected; control with infected animals without treatment; infected animals treated with fluconazole at 10 mg/kg/day; infected animals treated with CHT at 10 mg/kg/day; infected animals treated with CHT at 50 mg/kg/day. All treated groups received daily, intraperitoneally, CHT (10 or 50 mg/kg/day) or fluconazole (10 mg/kg/day) injection. The treatment was performed for 15 days and survival was monitored until the animals died. The CHT compound was diluted in polyethylene glycol 400 (20%), Tween 80 (0.05%) and PBS, and fluconazole was prepared in PBS.

After survival analysis another experiment was performed for colony-forming unit (CFU) recovery. Then, animals were infected and treated for 15 days with CHT or fluconazole (10 mg/kg/day). At the designated time end point, animals were anesthetized and euthanized. Brain and lung tissues were harvested to evaluate the fungal burden. The collected tissue was homogenized in PBS, serially diluted, and plated on YPD to determine the number of CFUs [25]. Three independent experiments were carried out with six animals per group.

### Systemic candidiasis

Mouse model of systemic candidiasis was established according to a previously described method by Wong *et al*. [26], with modifications. The mice were injected via the tail vein with 30 μl cell suspension of *C. albicans* SC5314, resulting in 10^5^ yeasts per animal 3 h before the start of antifungal treatment. Treatments were given to the mice via intraperitoneal injection at 3, 8, 24, 36, 48, 60, 72 and 84 h after infection with fluconazole (10 mg/kg) or CHT (10 mg/kg; n = 6 animals/group). The CHT compound and fluconazole were diluted according to previously described for cryptococcosis *in vivo* assay. Two control groups with infected and not-infected animals were also used. All of the mice were sacrificed after 96 h, and the kidneys were removed, weighed and then homogenized in PBS, and the homogenates were serially diluted before plating on Sabouraud dextrose medium plates. The plates were incubated at 37°C for 48 h, and fungal burden was expressed as the ratio of CFU/g of kidney.

### Statistical analysis

Percent survival and killing curves of *G. mellonella* were plotted and statistical analysis was performed by the Kaplan–Meier test using GraphPad Prism statistical software (GraphPad Software, Inc., CA, USA). Statistical analysis of results of antibiofilm activity, inhibition of *C. albicans* adhesion and murine tests used Newman–Keuls Multiple and the Student's *t*-test using GraphPad Prism and p-value ≤ 0.05 was considered significant and results were expressed as mean ± standard error of the mean.

## Results

CHT has demonstrated inhibition against 24 isolates of *Cryptococcus* spp. [16]. We expanded this, finding that CHT is also efficacious against *C. albicans*. Susceptibility tests with *C. albicans* isolates resulted in MIC values ranging from 1 to 4 mg/l for most isolates, but two clinical isolates, UFMG-CM-Y6080 and UFMG-CM-Y6073, did not showed susceptibility at concentrations up to 64 mg/l of CHT. Among six *C. albicans* clinical isolates examined, we observed fluconazole resistance with MIC values >64 mg/l (Table 1). Only one clinical strain of *C. albicans* exhibited dual resistance to both CHT and fluconazole. Sá *et al*. [27] showed evidence that CHT target action is related to interference in antioxidant fungal system and here in this work we show that in addition to this CHT is also capable of inhibiting *C. albicans* adhesion in  HBEC and shows an indication in reducing of biofilms.

**Table 1.** **Minimum inhibitory concentration of 2-(2-(cyclohexylmethylene)hydrazinyl)-4-phenylthiazole and fluconazole against several clinical isolate of *C. albicans.***

**Fungal species**	**Strains**	**MIC (mg/l) of *Candida* strains**	

		**CHT**	**FLZ**
*Candida albicans*	SC5314	4	1

*C. albicans*	ATCC 18804	2	2

*C. albicans*	UFMG-CM-Y6071	4	>64

*C. albicans*	UFMG-CM-Y6072	2	8

*C. albicans*	UFMG-CM-Y6073	64	4

*C. albicans*	UFMG-CM-Y6074	2	2

*C. albicans*	UFMG-CM-Y6075	1	8

*C. albicans*	UFMG-CM-Y6076	4	>64

*C. albicans*	UFMG-CM-Y6077	4	>64

*C. albicans*	UFMG-CM-Y6080	>64	>64

*C. albicans*	UFMG-CM-Y6081	2	>64

*C. albicans*	UFMG-CM-Y6082	2	>64

CHT: 2-(2-(cyclohexylmethylene)hydrazinyl)-4-phenylthiazole; FLZ: Fluconazole; MIC: Minimal inhibitory concentration

**Figure 2.**
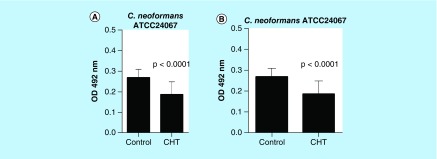
**Antifungal activity of 2-(2-(cyclohexylmethylene)hydrazinyl)-4-phenylthiazole in biofilms formed by *Cryptococcus* spp. by reduction assay with 2,3-bis(2-methoxy-4-nitro-5-sulfophenyl)-5-((phenylamino)carbonyl)-2H-tetrazolium hydroxide.** **(A)** *Cryptococcus gattii* L27/01. **(B)** *Cryptococcus neoformans* ATCC24067. Statistical analyses were performed by Student's *t*-test. Results were expressed as mean ± standard error of the mean.

Although we knew that CHT could inhibit planktonic cells as assessed by our previous observation of the low MIC of 1 mg/l found against *Cryptococcus* spp. [16], we interrogated the ability of CHT to inhibit biofilm formation. In order to verify the ability of CHT to inhibit the biofilm states sessile cells in biofilms formed by *C. gattii* L27/01 and *C. neoformans* ATCC24067 were challenged. Results of these experiments showed a significant reduction of the metabolic activity of yeasts in biofilms formed by *C. gattii* (44.2%) and *C. neoformans* (30.8%) after treatment with CHT compared with control biofilm without treatment (p < 0.0001 to both) (Figure 2).

**Figure 3.**
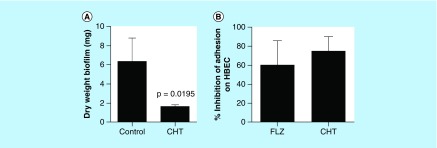
**Evaluation of the inhibition of *Candida albicans* virulence factors after treatment with 2-(2-(cyclohexylmethylene)hydrazinyl)-4-phenylthiazole at MIC.** **(A)** Evaluation of the reduction in biomass of biofilm formed by *C. albicans* SC5314 after treatment with CHT. Statistical analyses were performed by Newman–Keuls multiple comparison test. **(B)** Percent inhibition of *C. albicans* adhesion treated for 1 h with CHT and fluconazole at MIC on human buccal epithelial cells. Data representative of two independent experiments with 12 isolates of *C. albicans*. The control group without treatment represents 100% of adhesion of yeasts on human buccal epithelial cells (0% of inhibition). There were no significant differences between fluconazole or CHT treatment (p > 0.05). Statistical analysis was performed by Newman–Keuls multiple comparison test. Results were expressed as mean ± standard error of the mean. CHT: 2-(2-(cyclohexylmethylene)hydrazinyl)-4-phenylthiazole; FLZ: Fluconazole; HBEC: Human buccal epithelial cells.

Since CHT was effective in reducing Cryptococcal biofilms, we further investigated the capacity to inhibit *Candida* biofilm. We found a reduction in dry weight of *C. albicans* biofilm (73.8%) after treatment with CHT (Figure 3A), with a significant difference between control and treatment (p = 0.0195). Successful biofilm formation is dependent upon the capacity for cell adhesion in order to anchor fungi to a device or tissue. One of the most impacted sites by biofilm formation is the oral cavity [28,29]. The inclusion of CHT resulted in a 75.12 ± 12% reduction in *C. albicans* to HBEC (Figure 3B). The reduction of adhesion capacity was similar for CHT and fluconazole (60.5 ± 19%) treatment. The culmination of biofilm disturbance suggests that CHT can inhibit biofilm formation, a normally effective fungal strategy of invading a host and evading therapeutic damage.

**Figure 4.**
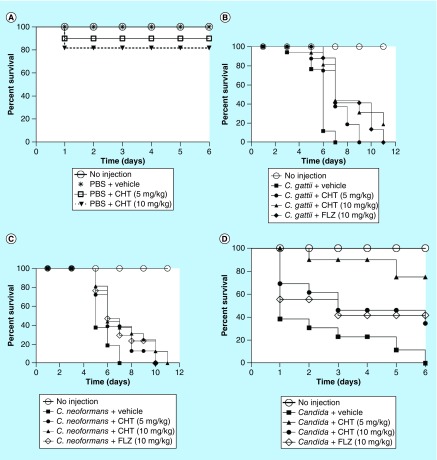
**Survival curve of the *Galleria mellonella* infected with *Cryptococcus gattii* L27/01 and *Cryptococcus neoformans* ATCC24067 and treated with 2-(2-(cyclohexylmethylene) hydrazinyl)-4-phenylthiazole.** **(A)** Evaluation of toxicity of CHT in *G. mellonella* larvae (p = 0.2059 to 5 mg/kg and p = 0.0820 to 10 mg/kg). **(B)** *G. mellonella* infected by *C. gattii* and treated with CHT at 5 mg/kg (p = 0.0001) and at 10 mg/kg (p = 0.0005), and FLZ (p = 0.0001). **(C)** *G. mellonella* infected by *C. neoformans* and treated with CHT at 5 mg/kg (p = 0.0042) and at 10 mg/kg (p = 0.007), and FLZ at 10 mg/kg (p = 0.0089). **(D)** Survival curve of the *G. mellonella* infected with *Candida albicans* SC5314 and treated with CHT. *G. mellonella* infected by *C. albicans* and treated with CHT at 5 mg/kg (p = 0.0002) and at 10 mg/kg (p = 0.0407), and FLZ (p =0.0059). Statistical analyses were performed by Kaplan–Meier. CHT: 2-(2-(cyclohexylmethylene) hydrazinyl)-4-phenylthiazole; FLZ: Fluconazole; PBS: Phosphate-buffered saline.

Based on these *in vitro* findings, we studied the efficacy of CHT *in vivo*. *G. mellonella* was selected as an invertebrate infection model for its susceptibility to various fungal pathogens [30]. The toxicity of the compound was tested in *G. mellonella* larvae and we found the LD_50_ >10 mg/kg in the absence of infection, thus, no significant toxicity of this thiazole derivative in the larvae (Figure 4A). After treatments with CHT, *G. mellonella* larvae infected with *C. gattii* L27/01 or *C. neoformans* ATCC24067 exhibited significantly prolonged survival (Figure 4C).

A *G. mellonella* infection model was also used to test the therapeutic potential against *Candida.* The CHT compound showed significantly prolonged larval survival (Figure 4D). The two doses tested were efficient, but the best dose was 5 mg/kg resulting in >70% of survival after 6 days of infection, while, in the same period, a dose equal to 10 mg/kg provided only 40% larval survival. Thus the broad-spectrum antifungal character of CHT is legitimized in an *in vivo* infection model.

**Figure 5.**
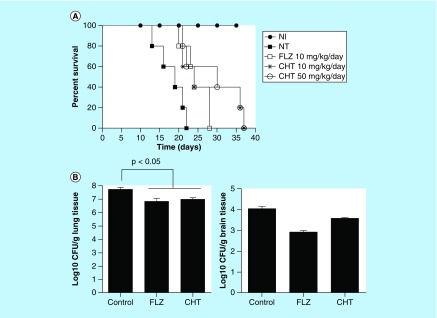
**Evaluation of 2-(2-(cyclohexylmethylene) hydrazinyl)-4-phenylthiazole treatment in murine model.** **(A)** Survival curve of the male C57BL/6 mice infected with *Cryptococcus gattii* L27/01 (10^5^ yeast) and treated with CHT and FLZ. The three treatments significantly increased survival at p < 0.05 to FLZ 10 mg/kg/day, CHT at 10 and 50 mg/kg/day (Kaplan–Meier test). Representative data from two independent experiments with n = 5. **(B)** Recovery of colony-forming units from lung and brain from male C57BL/6 mice infected with *C. gattii* L27/01 (10^5^ yeast) and treated CHT and FLZ for 15 days. There was a significant reduction in colony-forming units for the lung (p < 0.05), but not in the brain (p > 0.05) for both treatments. Statistical analyses were performed by Newman–Keuls multiple comparison test. Results were expressed as mean ± standard error of the mean. CFU: Colony-forming unit; CHT: 2-(2-(cyclohexylmethylene)hydrazinyl)-4-phenylthiazole; FLZ: Fluconazole; NI: Control not infected; NT: Control with infected animals without treatment.

The encouraging results from the insect model prompted us to extend our evaluation into a mammalian infection model. In a murine model of cryptococcosis caused by *C. gattii* the groups of animals infected and treated with fluconazole and CHT at 10 and 50 mg/kg/day exhibited a longer survival in comparison to control animals without treatment (p < 0.05) (Figure 5A). It was observed that there were still live animals up to day 37 of CHT treatment and in the fluconazole group all animals had died by day 28, whereas in the group without treatment, all animals expired before the 22nd day of the experiment. There was no significant difference between the two tested CHT concentrations. There was also no significant difference between CHT or fluconazole treatments.

**Figure 6.**
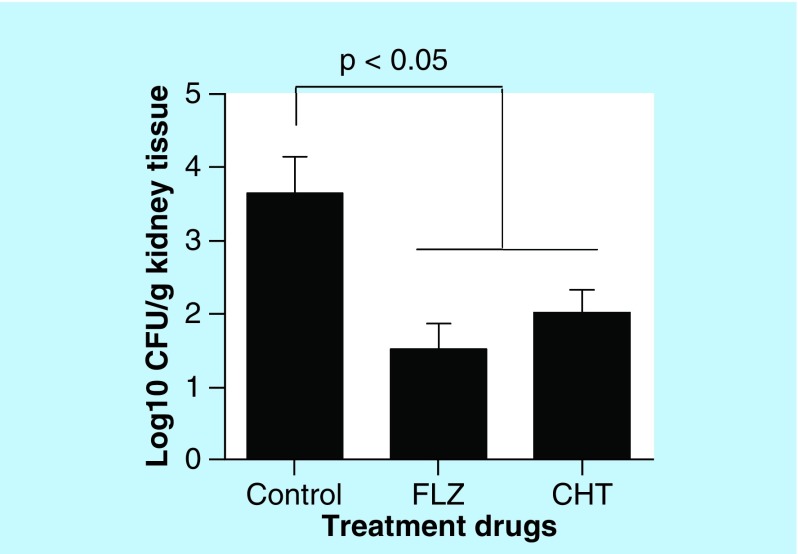
**Evaluation of 2-(2-(cyclohexylmethylene)hydrazinyl)-4-phenylthiazole treatment in murine model of systemic candidiasis.** Recovery of colony-forming units from kidneys from female C57BL/6 mice infected with *Candida albicans* SC5314 (10^5^ yeasts) and treated 2-(2-(cyclohexylmethylene)hydrazinyl)-4-phenylthiazole and fluconazole. Statistical analyses were performed by Newman–Keuls multiple comparison test. Results were expressed as mean ± standard error of the mean. CFU: Colony-forming unit; CHT: 2-(2-(cyclohexylmethylene)hydrazinyl)-4-phenylthiazole; FLZ: Fluconazole.

As there were no significant differences between the two doses of CHT tested, other animals were treated only at the dose of 10 mg/kg/day over 15 days to evaluate difference in fungal burden to the tissue. Both CHT and fluconazole treatment significantly reduced the amount of CFU recovered from the lung (p < 0.05) (Figure 5B). However, in the brain we observed only a small reduction in the number of recovered CFUs, not significant (p > 0.05). In murine model of systemic candidiasis the fungal burden of the kidneys were lower in mice that received treatment with CHT or fluconazole than in mice that not received treatment (Figure 6).

## Discussion

The heterocyclic thiazole CHT was previously evaluated in *in vitro* assays by Sá *et al*. [16] and showed interesting results against *C. gattii* and *C. neoformans*. In the present work we expand the fungal pathogens susceptible to the compound to include *C. albicans*. Our interrogation was able to correlate *in vitro* activity with the efficacy of CHT treatment in animal models.

In the biofilm assays with *Cryptococcal* species and *C. albicans* we observed that CHT compound were also active against sessile cells aggregated as biofilms formed by these species, which is a key factor in fungal pathogenesis [31]. The biofilm formation phases are very similar between *Cryptococcus* spp. and *C. albicans* [32]. Biofilms represent the most common mode of growth of microorganisms in nature, a state that presumably allows yeasts cells to both survive hostile environments, making the cells more resistant to host immune mechanisms and drug therapy [33].

*C. albicans* biofilm is formed through coordinated phases such as early, intermediate and maturation stages [32]. Especially in early growth phase yeasts need to be strongly adhered to the surfaces. Then, we observed that CHT treatment also interferes with the adhesion capacity of *Candida* yeasts to HBEC, which is closely related to biofilm construction. Adhesion of *C. albicans* to host cells is essential to colonization, biofilm formation and survival in the host, and this process requires the interaction of *Candida* adhesins with host cell receptors [34].

In view of this promising *in vitro* activity of the CHT compound we tested it in *in vivo* models. Initially we evaluated CHT in the invertebrate model with *G. mellonella* infected with *C. gattii, C. neoformans* or *C. albicans.* Treatment of larvae with CHT resulted in increased larval survival. Based on our results we believe that the CHT compound initiates some accumulation of harmful events on fungal cell that causes a failure in protecting the fungi from executing the virulence factors, such as *C. albicans* adhesion (which may be related to the *C. albicans* biofilm development).

In a previous work [16] we had demonstrated that there was no toxicity to mammalian cells, and recently we verified that CHT is nontoxic to human erythrocytes [27]. These findings indicate that CHT has potential in a murine model, which is considered the gold standard for studying pathogenesis, quantifying virulence, and analyzing the efficacy of antifungal drugs [30].

Thus, in the present work we observed that the CHT compound was efficient in prolonging the survival of mice infected with *C. gattii* L27/01 and *C. albicans* SC5314 similarly to fluconazole. The isolate L27/01 of *C. gattii* presents an intermediate virulence phenotype in mice and has been used in several types of studies in murine cryptococcosis model [25,30,35]. The reference isolate SC5314 of *C. albicans* presents high virulence and has been used in several works [36,37]. Jaen-Chen *et al*. [36] showed that intravenous injection of SC5314 results in mortality of animals between 8 and 35 days postinfection according to inoculum dosage.

Santos *et al*. [38] showed that C57BL/6 mice infected with L27/01 treated with fluconazole at 10 mg/kg/day had a significant increase in survival similar to the dose of 75 mg/kg/day. In addition to promoting a significant reduction in lung fungal load, which can be correlated with our work, confirming that the dose of 10 mg/kg/day is sufficient to generate significant effects and therefore can be used for comparison with experimental compounds.

The intravenous infection for systemic candidiasis is well characterized and reproducible model, and allows yeasts to spread rapidly throughout the body of the host; however, usually the infection is controlled in many organs but progresses in the kidneys in the absence of efficient antifungal treatment [39]. In the present study we observed similar control of fungal burden from kidneys after treatments with CHT and fluconazole, which is used for the treatment of systemic candidiasis.

## Conclusion

In the present work the efficiency of a new phenylthiazole was associated with important fungicidal activity, ability to inhibit virulence factors via inhibition of *C. albicans* adhesion in human buccal epithelial cell, and showed evidence of reduced *C. albicans* and *Cryptococcus* biofilms. We believe that all these processes observed *in vitro* are closely related to the significant results observed in the murine model of cryptococcosis and candidiasis. This study presents evidence of CHT's therapeutic potential.

## Future perspective

The substance presented in this study has potential for systemic use in the treatment of cryptococcosis and candidiasis, and also in topical use for oral candidiasis. The preliminary results reinforce the possibility that this compound, after all the necessary tests and clinical trials, could be used as a prototype for the development of a new antifungal.

**Executive summary**

- 2-(2-(cyclohexylmethylene)hydrazinyl)-4-phenylthiazole (CHT) is a thiazole derivative.
- CHT is active against *Candida* and *Cryptococcus*.
- In a previous study the mechanism of action of CHT was related open in the fungal antioxidant system.
- CHT works well in both suspension and biofilm cells.
- In *Candida*, CHT inhibits adhesion ability.
- In *Galleria mellonella*, treatment with CHT was effective for candidiasis and cryptococcosis model.
- In systemic use, CHT is efficient for the treatment of cryptococcosis and candidiasis in a murine model.
- CHT has shown activity against *Candida albicans* against cells in suspension and also in biofilm, besides affecting the ability of these yeasts to adhere to oral cells.
- CHT was also active in biofilms of *Cryptococcus gattii* and *Cryptococcus neoformans*.
- The experimental compound increased the survival of *Galleria mellonella* larvae infected with *C. albicans*, *C. gattii* and *C. neoformans*.
- In the murine model, CHT was able to reduce the fungal load of the lungs of mice infected with *C. gattii*.
- CHT was also efficient in reducing the fungal load in the kidneys of animals with systemic candidiasis.
